# A Retrospective Analysis of the Treatment Approach to Immune Thrombocytopenia in the Real World

**DOI:** 10.7759/cureus.5894

**Published:** 2019-10-11

**Authors:** Diana M Cuervo, Leonardo Enciso

**Affiliations:** 1 Internal Medicine, Universidad De La Sabana, Chia, COL; 2 Hematology, National Cancer Institute, Bogotá, COL

**Keywords:** immune thrombocytopenia, steroids, intravenous immunoglobulin

## Abstract

Introduction

Immune thrombocytopenia (ITP) is an acquired cause of thrombocytopenia in both the adult and children populations due to the accelerated destruction of platelets and/or suppressed platelet production. We present a retrospective analysis of a case series of patients in a single teaching institution with the objective of describing the clinical characteristics and different treatment approaches of patients with ITP.

Methods

A review of electronic health records was performed in the University Hospital Samaritana, Bogota, for inpatients between 2013 and 2016. Data were extracted for the patients with an ITP diagnosis for variables previously chosen and reviewed for descriptive analysis.

Results

A total of 55 patients were diagnosed with ITP; of these, 67.3% were female and the median age of this group of patients was 48 years. The majority presented with severe thrombocytopenia with 80% of patients having platelets less than 30000/µL. Of the 55 patients with a final diagnosis of ITP, only 54 received dexamethasone, methylprednisolone, or prednisone as the first-line treatment. The increment in platelet count after seven days of treatment was greater in the group treated with dexamethasone.

Conclusion

The diagnosis of ITP is of exclusion, there is no gold standard test, however, as it was shown in our results, various unnecessary studies are performed that increase costs during the diagnostic approach. Evidence supports that treatment with high-dose dexamethasone is associated with faster short- and greater long-term efficacy as compared to other steroids, however, it is not always the first choice in real-world patients. It is our belief that the implementation of a guideline will reduce testing and costs, and ensure better treatment options for our patient population.

## Introduction

Immune thrombocytopenia (ITP) is an acquired cause of thrombocytopenia in both the adult and children populations due to the accelerated destruction of platelets and/or suppressed platelet production [1-2]. Although it has been categorized as a rare disorder, with an incidence calculated in adults of 3.3/100000 per year and a prevalence of 9.5 per 100000 adults, it is a common diagnosis in hematology hospital consultations [1,3].

Since the 1950s, it is recognized that ITP is due to an autoantibody, mostly immunoglobulin G (IgG) directed against glycoprotein (GP) IIb/IIIA and GP Ib/IX that mediates platelet clearance [2]. However, around 30%-40% of patients have no detectable specific autoantibodies [1-2]. Antigen-presenting cells (APCs) can also present platelet antigens associated with major histocompatibility complex (MHC) class I molecules to CD8+T cells and activate cytotoxic T lymphocytes, which could also potentially impair platelet production. There is an immune dysregulation with a decreased regulatory T-cell population that can lead to a loss of tolerance, increase the secretion of IL-2 and IFN-g, and promote B-cell differentiation [1-2].

With a better understanding of immunopathogenesis and the development of new treatment options, there has been a rapid development of guidelines and clinical recommendations in the last 20 years; and with that, different terms and definitions to refer to this hematologic disorder. Current evidence continues to hold steroids and intravenous immunoglobulin as first-line treatment. However, these options are not curative and are associated with adverse events, hence second-line treatment has been developed, including rituximab, thrombopoietin receptor agonists, such as romiplostim and eltrombopag, and immunosuppressive agents. These second-line agents have been associated to reduce morbidity [4].

In Colombia, the incidence rate of ITP has not been determined, three single-center retrospective case series have been published [5-7]. The largest study of a cohort in a teaching hospital by Palmezano-Diaz J et al. calculated a local prevalence of 33 cases per 100000 patients. It is believed that due to the unknown epidemiology and clinical importance in our country, there are no national guidelines for the diagnostic approach or treatment of ITP [5]. This leads to internal medicine and hematologist specialties to guide treatment options by varied international literature.

Here, we present a retrospective analysis of a case series of patients with an initial presentation of thrombocytopenia in a single teaching institution, with the objective of describing the clinical characteristics and different treatment approaches of patients with a final diagnosis of ITP. This study aims to stimulate more research in our population and develop national guidelines.

## Materials and methods

A review of electronic health records was performed for all adult inpatients admitted, with thrombocytopenia as their primary or secondary diagnosis in terms of the International Classification of Diseases, 10th revision (ICD-10), from 2013 to 2016 at the University Hospital Samaritana, Bogotá. Data extraction from the chart review was performed only for patients with a confirmed diagnosis of ITP. The study was presented and approved by the ethics committee of the hospital.

Patient selection

Electronic health records between 2013 and 2016 were filtered to select only patients with a diagnosis of thrombocytopenia based on the ICD-10 classification as D694 other primary thrombocytopenia, D695 secondary thrombocytopenia, D696 thrombocytopenia or unspecified, and D693 idiopathic thrombocytopenic purpura.

The following information was extracted from the charts reviewed for each eligible patient with the diagnosis of ITP: age, sex, date of hospitalization, initial complete blood count, major bleeding, direct antiglobulin test, antinuclear antibodies and specific antibodies for secondary autoimmune disease, serology for viral etiologies, initial and follow-up treatment strategies, and severe infection related to treatment. The final outcome for every patient was also recorded.

Statistical analysis

Quantitative values are reported as mean and median (first-third interquartile) and qualitative data as percentages. P-values were derived using Fisher’s exact test and the Mann-Whitney test for qualitative and quantitative values, respectively. Analysis of variance (ANOVA) was used to compare platelet counts between the groups of first-line treatment, and it was verified by the Tukey test. P<0.05 was considered significant. Statistical analyses were performed with STATATM/SE 15.1 Software (StataCorp LLC, Texas, US).

## Results

Of the patients admitted to the University Hospital Samaritana between 2013 and 2016, a total of 55 patients were diagnosed with ITP; of those, 67.3% were female and the median age was 48 years old, the majority being younger than 60 years old (Table 1). No statistical difference was found between age and sex calculated (P = 0.44 Wilcoxon rank-sum). The majority of patients presented with severe thrombocytopenia, with 80% of patients having a platelet count of less than 30000/µL. As shown in Table 1, the median platelet count at presentation was 11000/µL with an interval between 0 and 79000/µL. No statistical difference was found when discriminated by sex (P = 0.38 Wilcoxon rank-sum). Other hematologic parameters are also shown in Table 1. Information on bleeding events at presentation was available in 52 out of 55 patients; major bleeding was documented in three (5.77%) patients and no statistical difference was found when discriminated by initial platelet count (P = 0.42 Wilcoxon rank-sum).

**Table 1:** Initial characteristics of patients diagnosed with ITP ITP: immune thrombocytopenia; VPM: mean platelet volume; fL: femtolitre; Ɨ Information was available for 52 patients; ƗƗ Information was available for 46 patients; NS: no statistical significance in terms of initial platelet count

			Total population n = 55	
Female n(%)			37 (67.3%)		
Age years median (range)			48 ( 17, 89)		
Hematologic characteristics median (range)					
	Leukocytes (/µL)		9090 (2750, 36920)		
	Neutrophils (/µL)		6650 (1490,31101)		
	Hemoglobin (g/dL)		13.6 (4.2, 17.2)		
	Platelets (/µL)		11000 (0, 79000)		
	VPM (fL)		9.9 (0, 13.6)		
Ɨ Major bleeding at presentation n(%)			3 (5.77%)		NS
ƗƗ Bone marrow biopsy performed n (%)			16 (34.78)		NS

Additional testing was performed variably in patients. Of the 55 patients, the direct anti-globulin test was available in 19, with a positive result in five (26%). The antinuclear antibody was available for 32 patients, with a positive high titer (>1/160) in four patients (12.5%). No patients were found to have antiphospholipid syndrome concomitantly. No viral infection was prevalent. Twenty-four out of the 55 patients were tested for human immunodeficiency virus (HIV), which was positive in one patient. The complete infectious profile is presented in Table 2. The cases that were considered ITP secondary to infection were the minority but were included because the first-line treatment was used as the primary therapeutic approach. No additional testing was done, except for a bone marrow biopsy, which was performed in 16 patients. No statistical difference was found between age and the decision for a bone marrow biopsy in these cases (P = 0.53 Wilcoxon rank-sum test).

**Table 2:** Immunological and infectious profile in patients with ITP ITP: immune thrombocytopenia; Ig: immunoglobulin; HIV: human immunodeficiency virus; ELISA: enzyme-linked immunosorbent assay

			Positivity n (%)
Direct antiglobulin test n = 19			5 (26.3)
High titer (>160) antinuclear antibody n = 32			4 (12.5)
IgG antiphospholipid n = 10			1 (10)
IgM antiphospholipid n = 8			2 (25)
IgG anticardiolipins n = 19			1 (5.3)
IgM anticardiolipins n = 19			1 (5.3)
IgG β2 glycoprotein n = 7			1 (14.3)
IgM β2 glycoprotein n = 5			0
Lupic anticoagulant n = 11			5 (45.5)
HIV ELISA n = 24			1 (4.2)
Hepatitis C antibodies n = 41			0
Hepatitis B superficial antigen n = 42			1 (2.4)

Treatment information was available for all patients, as shown in Table 3. Of the total population, 54 patients received first-line treatment with steroids, dexamethasone (61%), methylprednisolone (20.5%), and prednisolone (18.5%); one patient with previously treated chronic ITP received an agonist of thrombopoietin. The preferred treatment for the group of patients with a lower median platelet count was intravenous steroids versus oral. The median platelet count was 7000/µL, 11000/µL, and 28500/µL in groups of dexamethasone, methylprednisolone, and prednisolone, respectively. Comparing the initial platelet counts between the three treatment groups, a statistical difference was found between the groups treated with prednisolone vs. dexamethasone (P = 0.006, CI (4956 - 34255) Tukey test).

**Table 3:** First-line treatment differences in terms of initial platelet count (/µL) and response after seven days and at discharge

			Total population treated = 54
Dexamethasone n = 33			
	Initial median platelet count (IQR)		7000 (13000)
	Mean platelet count increase after 7 days of treatment (SD)		72255 (81364)
	Mean platelet count at discharge (SD)		105546 (71400)
Methylprednisolone n = 11			
	Initial median platelet count (IQR)		11000 (22100)
	Mean platelet count increase after 7 days of treatment (SD)		33357 (48076)
	Mean platelet count at discharge (SD)		72490 (49063)
Prednisolone n = 10			
	Initial median platelet count (IQR)		28500 (42000)
	Mean platelet count increase after 7 days of treatment (SD)		32000 (8185)
	Mean platelet count at discharge (SD)		66714 (65260)

The change of platelet count after seven days was available in 30 patients out of the 55 patients. The mean increase in platelet count after seven days of treatment was greater in the dexamethasone group (72255/µL) as compared to the other two groups. However, this difference is not statistically significant (P = 0.38 ANOVA), though it could be the effect of the reduction in the sample population. The information for platelet count at discharge was available in 47 out of 55 patients due to restrictions with the electronic charts. The mean platelet count at discharge was greater in the treatment group of dexamethasone (105546/µL) as compared to the methylprednisolone (72490/µL) and prednisolone (66714/µL) groups. No statistical difference exists between this comparison (P = 0.10 ANOVA). The data to evaluate the proportion of patients that achieved platelet counts greater than 30000/µL was available in 30 out of the 55 patients. The proportion of patients that achieved platelet counts at discharge of more than 30000/µL was 12 (60%) in the group treated with dexamethasone, four (57%) in the group treated with methylprednisolone, and two (66.7%) in the prednisolone group; no statistical difference was found (P = 1 Fisher’s exact test).

Information about second-line treatment strategies was available. Intravenous immunoglobulin (IVIg) as initial treatment concomitantly with steroids was used in 14 patients, the range of the initial platelet count of these patients was 0-28000, with a median of 3500/µL. Two patients had previously been diagnosed with ITP and were in treatment with romiplostim, which is why this therapy was also initiated. Additionally, five patients received treatment with rituximab, nine patients with eltrombopag, and six patients with romiplostim. Splenectomy was a treatment option for six patients.

Of the 52 patients with registered information, a severe infection occurred during the first hospitalization in four (7.7%) patients; no statistical difference was found (P = 0.17 Fisher's exact test). In the three-year period, mortality was documented in five patients. 

## Discussion

Immune thrombocytopenia defined as an acquired immunological cause of thrombocytopenia diagnosed with a platelet count of <100000/µL. It is important to understand the full extent of this pathology, as it is frequently encountered in everyday life. This disease has been described in children as self-limited and in adults as a chronic disease, with a peak incidence in childhood and in young adults and the elderly [1]. Our study did not include a childhood population because our institution only admits adult patients. The epidemiology varies between different publications, calculated as an incidence for primary ITP 3.3/100 000 adults per year and a prevalence of 9.5 per 100000 adults [1]. In our country, the incidence and prevalence have not been calculated till the date of this publication. During the three-year observation period in the teaching hospital, we found a total of 369 in-patients with a diagnosis of thrombocytopenia based on the ICD-10 classification, with a total of 55 patients with a final diagnosis of immune thrombocytopenia. Recently, in our country, also in a teaching hospital, 128 cases were analyzed, including patients older than 13 years old; prevalence was calculated as 33 cases per 100000 patients [5]. We did not calculate the prevalence in our institution because we only included cases that required attention in the emergency room and follow-up in the general ward. We did not include patients with a stable disease during this period of time. 

In our study, out of the total of 55 patients with ITP, the majority (67.3%) were women, with a median age of 48 years and no age peak. There was no difference between age and gender in our population, neither with gender nor initial platelet count. Female predominance is similar to the three different case series in our country and the international literature [5-7]. In terms of age, the median is around 50-55 years old with a female predominance but after 60 years of age, the proportion between males and females is equal, and in other cases, men predominate with the older age population [1,8]. Major bleeding, defined by World Health Organization grades 3-4, was found in only three (5.77%) patients at presentation; no statistical difference was found between this event and the initial platelet count.

ITP is a diagnosis of exclusion in cases with isolated thrombocytopenia of less than 100000/µL [9], which was true with the majority of our cases with a median leukocyte and hemoglobin count within range. The median initial platelet count was 11000/µL and the range was 0-79000/µL; the higher number count was for patients with stable disease who were admitted to the hospital for a secondary reason. The median of the MPV was 9.9 fL and the range was 0-13.6 fL. This parameter is found to be increased in cases of ITP, with a sensitivity and specificity that ranges between 75% and 92.3%, is due to increased thrombopoiesis with young and large platelets [10]. We believe that the discordance of our results compared to the literature is because our automated cell counter is based on electrical impedance and cannot differentiate between platelets and other similar-sized particles [10].

Meillon-Garcia found that the direct antiglobulin test was requested always/frequently in only 52% of the time during the diagnosis of ITP [11]. In our patient population, the minority had positivity for a direct anti-globulin test (26.3%), and six patients had anemia associated. The International Working Group (IWG) recommends that the direct antiglobulin test is appropriate if anemia with a high reticulocyte count is found concomitantly and there is consideration of anti-D immunoglobulin for treatment [12]. A multi-institutional review in 311 children found a positive direct-antiglobulin test was independently associated with chronic ITP in a multivariate logistic regression model [13]. However, the clinical significance of this recommendation hasn’t been confirmed in adults.

No patients were found to have the antiphospholipid syndrome or another autoimmune disease concomitantly. In the most recent case series in our country with similar population characteristics, positivity for antinuclear antibodies was also found, at a low percentage (7.8%) of patients with ITP [5]. In patients with ITP, antiphospholipid antibodies are found in 26% of cases [14]. Indication for a further immunological profile has not been consistent with different publications. The presence of antinuclear antibodies, antiphospholipid antibodies, and lupus anticoagulant has been associated with thrombosis, which is why this profile has been given importance due to its role in defining prognosis [8]. However, a positive antinuclear antibody without the clinical manifestation of autoimmune disease is not associated with another disease [1]. As mentioned earlier, no test can confirm the diagnosis of ITP, it is a diagnosis of exclusion [15]. The 2011 American Society of Hematology evidence-based practice guideline does not recommend the routine use of antinuclear, antiphospholipid, and antiplatelet antibodies [15]. A national guideline has to be created to clearly indicate the cases necessary to obtain additional autoimmune profiles to reduce the cost of unnecessary exams and to avoid missing cases of secondary ITP.

There are also differences between the requests for the infectious panel. From the total study population, the human immunodeficiency virus (HIV) test was requested in 24 patients, hepatitis C virus (HCV) test in 41 patients, hepatitis B virus (HBV) test in only three patients. However, various international guidelines recommend testing for HIV, HCV, cytomegalovirus (CMV), and Helicobacter (H.) pylori [8,12,14]. The latter study was not performed in any patient in our population. It has been estimated that the prevalence of H. pylori in one center in Medellin, Colombia, was 36.4%; there is no information for the rest of the country [16]. Studies suggest the eradication of H. pylori plays a role in the treatment of ITP, even as first-line treatment [17-18]. Keeping in mind what is previously mentioned, it is necessary to determine the effect of H. pylori in our patient population in order to create recommendations for our population.

Bone marrow examination was performed in 16 patients; no statistical difference was found between the age of the patient and the decision to perform the procedure. This procedure is not necessary for diagnosis, however, it is recommended in patients over 65 years of age, with atypical features, abnormalities in blood count or film examination, presence of organomegaly, or those who do not respond well to steroids of IVIg [4]. The indications for bone marrow examination were not explored in our study.

The initial treatment in 54 patients was steroids; one patient did not receive treatment with steroids because he had chronic ITP, with treatment with romiplostim, and presented with an initial platelet count of 65000/µL without the manifestation of bleeding. IV steroid treatment was used in cases with a lower initial platelet count, dexamethasone and methylprednisolone with a median count of 7000/µL and 11000/µL, respectively, comparatively with prednisolone with a median count of 28500/µL. The comparison of the platelet counts between the groups treated with dexamethasone versus prednisolone was statistically different. It is important to notice that the absolute platelet count increment by day seven is greater in the group treated with dexamethasone as compared to the other two (Table 2). However, no statistical difference was found, probably due to the small number of the population. No difference was found between the treatment and the infections groups. The treatment regimen in this institution with high-dose steroids is to continue later, with 1 mg/kg/d of prednisolone with a tapering regimen decided by each individual hematologist. The duration of steroid treatment has not been established, however, it is known that there is a high relapse rate and the treatment duration should not be less than three weeks [8]. Nonetheless, the response rate does not improve with prolonged steroid treatment and there is an increased risk of side effects.

The platelet count response after seven days of treatment can correlate with the results of the GIMEMA (Gruppo Italiano Malattie EMatologiche dell'Adulto) multicenter study. In our study, after seven days of treatment with dexamethasone, the mean platelet count was 72200/µL, in the GIMEMA multicenter pilot study, the median platelet count after the fourth day of the first therapy cycle was 92000/µL [19]. Mazzucconi et al. reported an increase in overall response after each cycle, from 69.5% to 85.6% [19].

It has been agreed that the efficacy of pulsed corticosteroids is due to the reduction in the production of immunoglobulin by B cells, and its long life and low cost as its advantage [19]. Keeping that in mind, remembering the results of the GIMEMA trial and ratifying the effect of dexamethasone in the real-world experience, it is our belief that the high-dose dexamethasone protocol for treatment of ITP in four-cycle therapy with intervals of 14 days is a feasible treatment option for our study population due to its shorter duration, long-term efficacy, and reduction of steroid-related side effects.

IVIG is part of first-line treatment in various guidelines, a generally safe option that is used in cases of severe thrombocytopenia or acute bleeding [4]. This correlates in our population, used in only 14 patients with a median platelet count of 3500/µL; however, only 5.77% presented with major bleeding. This study did not evaluate lower grades of bleeding, which is the most frequent presentation in ITP, and as it is has been studied in other academic centers as one of the most important indications for the use of IV immunoglobulin [20].

Other forms of therapy, such as splenectomy, rituximab, and agonist of thrombopoietin receptors, were not used frequently, probably due to the fact that this teaching hospital is a reference center for emergency patients, but the outpatient follow-up is limited due to the different health insurance companies.

The study population was filtered from the health records using the ICD-10 classification as previously specified, though if patients were not classified as such in their health records with an established ITP diagnosis, these were not included in the analysis. The most important limitation to our study is missing data and loss to follow-up of patients, which limits the possibility to evaluate the treatment efficacy in the short and long terms. In spite of the limitations, this study offers a glimpse of the real-life approach and treatment of acute ITP in our country, which acts as an influence to develop guidelines to reduce costs and adverse effects and to improve overall efficacy.

## Conclusions

Clinical practice in the real world is lagging behind the information available for the approach to the diagnosis and treatment of ITP. There is a wide variety of approaches to the diagnosis of ITP, which increases costs due to unnecessary tests and other occasions that the recommended studies are not performed. In terms of treatment, high-dose dexamethasone has evidenced that is associated with long-term efficacy as compared to other steroids, however, it isn’t always the first choice in real-world patients. More information is needed in this population in terms of the use and efficacy of second-line therapies, evidence which is limited by health insurance companies. Local guidelines need to be developed to address these concerns and, in this way, continue research in our population.
